# Supplementing the Nuclear-Encoded PSII Subunit D1 Induces Dramatic Metabolic Reprogramming in Flag Leaves during Grain Filling in Rice

**DOI:** 10.3390/plants12163009

**Published:** 2023-08-21

**Authors:** Ai-Zhen Sun, Juan-Hua Chen, Xue-Qi Jin, Han Li, Fang-Qing Guo

**Affiliations:** 1 The National Key Laboratory of Plant Molecular Genetics and CAS Center for Excellence in Molecular Plant Sciences, Institute of Plant Physiology & Ecology, Chinese Academy of Sciences, Shanghai 200032, China; azsun@cemps.ac.cn (A.-Z.S.); jhchen02@sibs.ac.cn (J.-H.C.); jinxueqi@cemps.ac.cn (X.-Q.J.); lihan@cemps.ac.cn (H.L.); 2 University of Chinese Academy of Sciences, Beijing 100049, China

**Keywords:** metabolome profiling, D1, photosynthetic efficiency, glutamine, endogenous nitrogen source, flavonoids, rice

## Abstract

Our previous study has demonstrated that the nuclear-origin supplementation of the PSII core subunit D1 protein stimulates growth and increases grain yields in transgenic rice plants by enhancing photosynthetic efficiency. In this study, the underlying mechanisms have been explored regarding how the enhanced photosynthetic capacity affects metabolic activities in the transgenic plants of rice harboring the integrated transgene *RbcS^PTP^-OspsbA* cDNA, cloned from rice, under control of the *AtHsfA2* promoter and N-terminal fused with the plastid-transit peptide sequence (*PTP*) cloned from the *AtRbcS*. Here, a comparative metabolomic analysis was performed using LC-MS in flag leaves of the transgenic rice plants during the grain-filling stage. Critically, the dramatic reduction in the quantities of nucleotides and certain free amino acids was detected, suggesting that the increased photosynthetic assimilation and grain yield in the transgenic plants correlates with the reduced contents of free nucleotides and the amino acids such as glutamine and glutamic acid, which are cellular nitrogen sources. These results suggest that enhanced photosynthesis needs consuming more free nucleotides and nitrogen sources to support the increase in biomass and yields, as exhibited in transgenic rice plants. Unexpectedly, dramatic changes were measured in the contents of flavonoids in the flag leaves, suggesting that a tight and coordinated relationship exists between increasing photosynthetic assimilation and flavonoid biosynthesis. Consistent with the enhanced photosynthetic efficiency, the substantial increase was measured in the content of starch, which is the primary product of the Calvin–Benson cycle, in the transgenic rice plants under field growth conditions.

## 1. Introduction

As unavoidable reactive byproducts of photosynthesis, a variety of reactive oxygen species (ROS) are dynamically generated in the chloroplast, which can induce severe photo-oxidative damage of photosynthetic apparatus, especially for photosystem II (PSII), leading to the inhibition of photosynthesis termed photoinhibition [1,2,3,4,5,6]. Notably, among the core subunit proteins of PSII, the D1 protein, encoded using the chloroplast gene *psbA*, is the most susceptible target with respect to ROS attacks, and on the other hand, ROS can also repress the synthesis of D1 by inhibiting the translation of the *psbA* mRNA [3,5,6,7,8,9,10,11,12,13]. Critically, a physiological process, the so-called PSII repair cycle, has operated to repair the damages of PSII and thus alleviate the levels of photoinhibition, during which the damaged D1 protein is replaced with the de novo synthesized D1 [2,11,13].

On the other hand, it has been demonstrated that the D1 stability also plays a key role in maintaining photosynthetic efficiency in higher plants. In a recent study, loss-of-function of a novel rice regulatory protein PAP90 (PSII auxiliary protein similar to 90 kDa) led to instability of the D1 protein, causing inadequate accumulation of photosynthetic complexes, especially for PSII with severely inhibiting photosynthetic efficiency in rice [14]. In addition, transgenic tomato plants accumulating glycinebetaine exhibited heat-tolerant phenotypes due to preventing heat-induced photoinhibition by protecting the stability of the D1 protein with the accumulated glycinebetaine under heat stress [15]. Although D1 has long been thought to be a highly stress-sensitive component of the photosynthetic machinery that affects photosynthetic light reactions, enhancing photosynthetic efficiency based on manipulating the expression of the D1 protein is still challenging in most plant species, especially for crop plants, mainly due to restrictions from the technical bottlenecks in plastid transformation [16,17]. Our recent studies have demonstrated that nuclear-origin supplementation of the D1 protein by overexpressing the Arabidopsis chloroplast gene *psbA* in the nuclear genome significantly increases the photosynthetic assimilation rates, biomass, and grain yield in the transgenic lines of rice under fields as well as in the transgenic plants of Arabidopsis and tobacco under control growth conditions [18]. Our findings have provided a new strategy and feasible approach to manipulating the chloroplast gene in the nucleus for improving photosynthetic efficiency and increasing crop productivity under normal and heat-stress conditions without being restricted by the technical bottlenecks for doing plastid transformation in crops such as rice [17,19,20,21,22,23,24].

In this study, the metabolite profiles of flag leaves were characterized under field growth conditions in wild-type and the transgenic rice plants harboring the integrated transgene *RbcS^PTP^-OspsbA* cDNA under control of the *AtHsfA2* promoter, which is similar to the construct used in our previous study [18], except for the Arabidopsis chloroplast gene *psbA* being replaced with the rice counterpart. Consistently supplementing OsD1 significantly increases biomass and productivity in these newly generated transgenic lines of rice with enhanced photosynthetic efficiency. We performed a comparative metabolomic analysis to explore and characterize the patterns of metabolic reprogramming induced by the enhanced photosynthetic capacity as measured in the transgenic rice plants relative to the wild-type plants under field growth conditions. Metabolome profiling was performed using liquid chromatography-mass spectrometry (LC-MS), resulting in the identification of a total of 361 differential metabolites using the LC-MS analysis in the flag leaves of the transgenic rice plants. This study has been designed to validate the utility of combining photosynthetic traits and agronomic and metabolomic traits as an approach to evaluate potential improvement targets for future breeding selection and the assessment of useful traits in crop plants. 

## 2. Results

### 2.1. Supplementing OsD1 Increases Photosynthetic Efficiency, Biomass and Yield 

**Figure 1:**
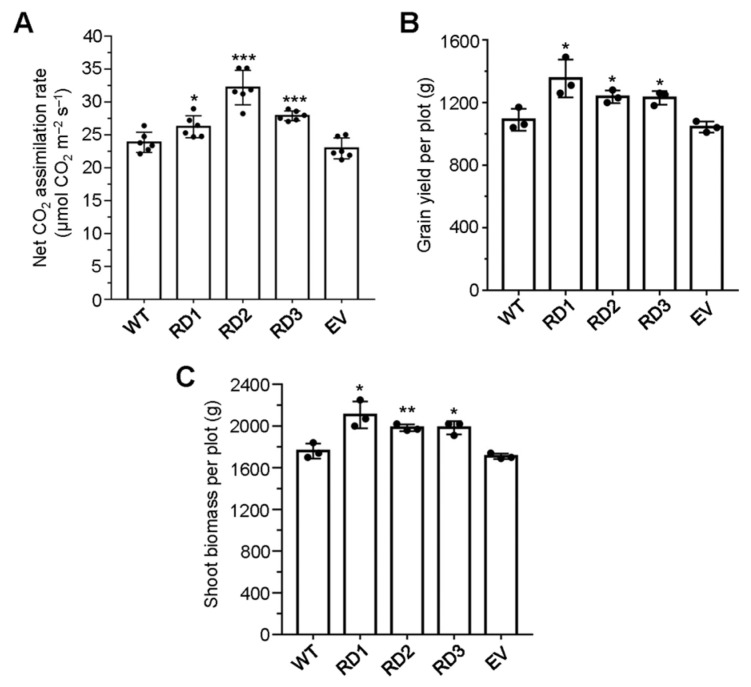
Supplementing OsD1 of nuclear origin increases the CO_2_ assimilation rate, biomass, and grain yield in rice. (**A**) Net CO_2_ assimilation rates were measured in wild-type (WT) and three transgenic lines of rice (RD1, RD2, and RD3) harboring the *pHsfA2::RbcS^PTP^-OspsbA* cDNA construct under field growth conditions (n = 6). Comparative analysis of grain yield per plot (**B**) and shoot biomass per plot (**C**) between WT and three transgenic lines of rice (RD1, RD2, and RD3) are shown (n = 3); 36 plants were planted in a plot. The transgenic rice line harboring the empty vector (EV) was taken as a negative control. Individual values (black-coded dots) and means are shown. Bars indicate the SD. * *p* < 0.05, ** *p* < 0.01, *** *p* < 0.001, two-sided Student’s *t*-test.

The transgenic rice plants expressing the integrated transgene *RbcS^PTP^-OspsbA* cDNA under the control of the AtHsfA2 promoter were generated. For targeting the chloroplast, the plastid-transit peptide sequence (*RbcS^PTP^)* was cloned from the *AtRbcS* as described [18], encoding the small subunit of ribulose 1,5-bisphosphate carboxylase–oxygenase (Rubisco), and then fused to the N terminus of the *OspsbA* cDNA. The expression levels of the integrated transgene and the abundance of the D1 protein in three transgenic lines (RD1, RD2, and RD3) were confirmed by performing quantitative RT-PCR and Western blots using the antibody against D1, respectively (Supplemental Figures S1 and S2A,B). The results showed that the abundance of D1 increased in three transgenic lines compared with the wild-type plants, which is consistent with the expression levels of *RbcS^PTP^-OspsbA* cDNA (Supplemental Figure S2A,B). With respect to the effects of supplementing the nuclear-encoded OsD1 on photosynthetic efficiency during grain filling, the photosynthetic carbon fixation rates increased significantly in all of three transgenic lines, RD1, RD2 and RD3 (Figure 1A). In addition, the grain yield of transgenic lines was remarkably increased by 24.1%, 13.5%, and 12.8% in the transgenic lines of RD1, RD2, and RD3, respectively, compared with the wild-type (WT) plants (Figure 1B) and the shoot biomass was significantly increased by 19.7%, 12.7% and 12.7% (Figure 1C). It should be noted that the transgenic line of rice containing the empty vector (EV) was taken as a negative control and exhibited slight decreases in all measurements, but no significant difference was detected when compared with the WT plants. These results indicate that supplementing OsD1 enhances photosynthetic efficiency and increases biomass and yield in rice.

### 2.2. Differential Metabolites Identified in the RD2 Transgenic Line by LC-MS

**Figure 2:**
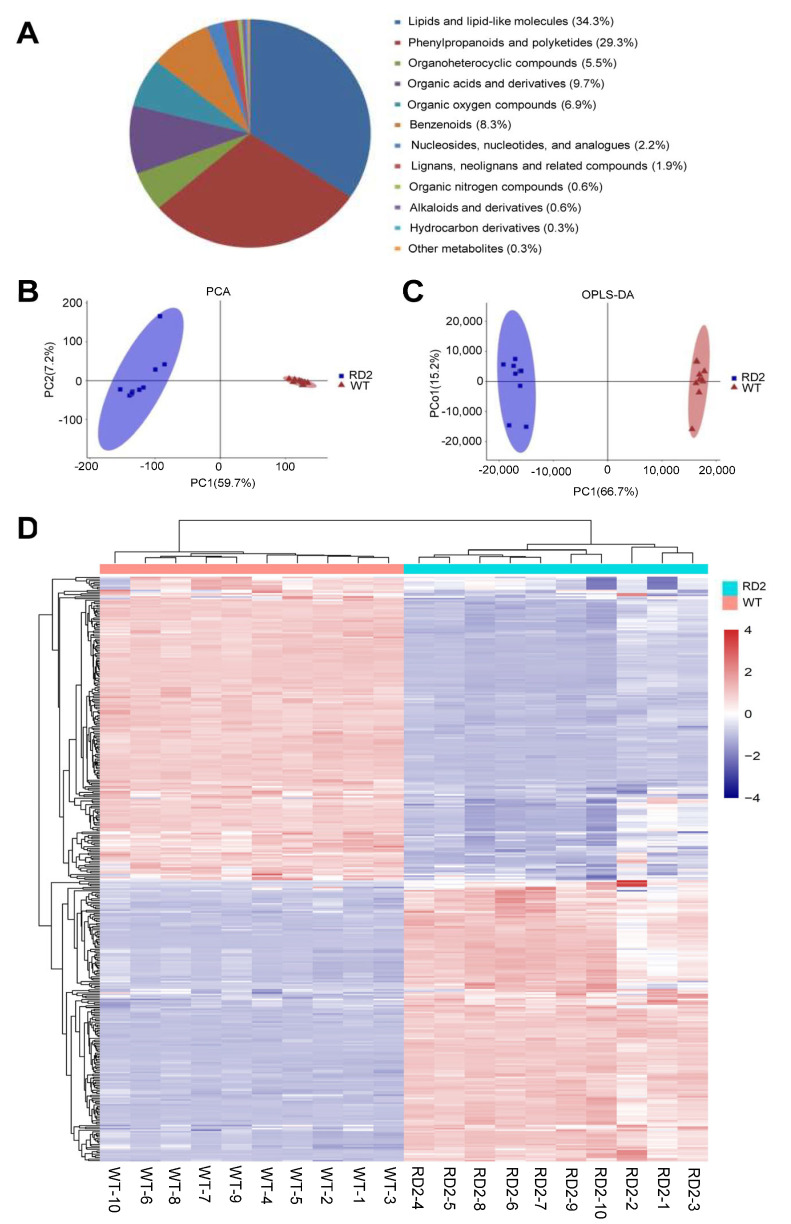
Metabolome profiling in the flag leaves of WT and the transgenic rice line RD2 by LC–MS. (**A**) Classification and proportion of a total of 361 metabolites differentially identified between WT and the transgenic rice line RD2 using the LC-MS analysis. (**B**) The plot of principal components analysis (PCA) score plot of 10 biological samples taken from the flag leaves of WT and the transgenic rice line RD2, respectively, using the LC-MS analysis. (**C**) Orthogonal partial least square discriminant analysis (OPLS-DA) score plot of 10 biological samples taken from the flag leaves of 10 groups of plants of WT and transgenic rice line RD2 using the LC-MS analysis. (**D**) Overview of the annotated metabolites and heatmap of hierarchical cluster analysis (HCA) from 10 biological samples of WT and transgenic rice line RD2 using the LC-MS analysis. Analysis was conducted with an average of ten independent biological replicates. The relative peak area was normalized by the average value of all samples. Fold changes are visualized using a color gradient from red (high) to blue (low).

To explore the comprehensive variations in metabolites between WT and the transgenic line RD2, the different metabolites in flag leaves of two genotypes during the grain-filling stage were analyzed and filtered via metabonomic analysis. A total of 361 differentially detected metabolites were identified using LC-MS based on VIP >1, *p*-value < 0.05. These differentially detected metabolites were divided into 12 classes, including lipids and lipid-like molecules (34.3%), phenylpropanoids and polyketides (29.3%), organoheterocyclic compounds (5.5%), organic acids and derivatives (9.7%), organic oxygen compounds (6.9%), benzenoids (8.3%), nucleosides, nucleotides, and analogs (2.2%), lignans, neolignans and related compounds (1.9%), organic nitrogen compounds (0.6%), alkaloids and derivatives (0.6%), hydrocarbon derivatives (0.3%) and other compounds (0.3%) (Figure 2A).

The principle component analysis (PCA) was used to observe the overall distribution among the samples and the stability of the whole analysis process. As shown in Figure 2B, the first principal component (PC1) could explain 59.7% of the total variance and separate samples from the two groups (Figure 2B). The second principal component (PC2) could explain 7.2% of the total variance. The samples with ten biological replicates were gathered into each group and were clustered to the same side along PC1 and completely distinguished from each other. The score figure in the orthogonal partial least square discriminant analysis (OPLS-DA) confirmed that the metabolites detected in the flag leaves showed a significant variation between WT and RD2 and were divided into two groups (Figure 2C). Samples of each group were gathered in a certain area. The classification results were consistent with those of PCA, and the score plot clearly showed distinct differences in metabolites between WT and RD2 plants (Figure 2B,C). These results indicate that enhanced photosynthesis by supplementing OsD1 induces dramatic metabolic reprogramming in the transgenic plants of RD2. The heatmap of log2FC was applied to screen the differentially identified metabolites in the group of the RD2 plants when compared with the WT plants, and the screened metabolites were hierarchically clustered into two main branches, suggesting that the analysis was stable and repeatable and displayed an obvious variation in total metabolites between two groups (Figure 2D).

### 2.3. Alterations in the Amino Acid and Nucleotide Metabolites Identified in the RD2 Transgenic Line 

**Figure 3:**
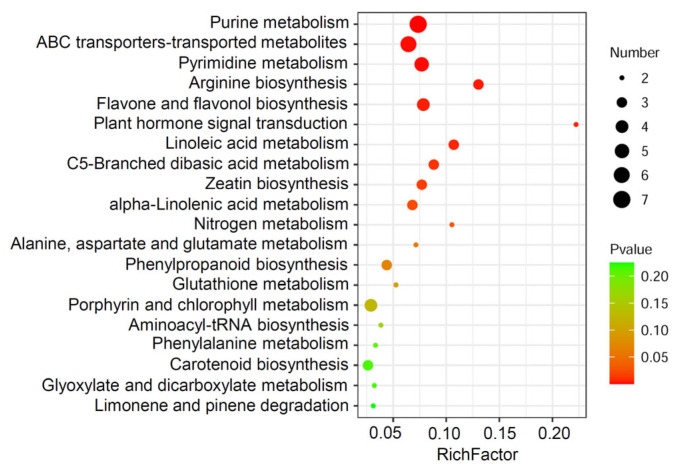
KEGG pathway enrichment of differential metabolites identified in the flag leaves of WT and transgenic rice line RD2 using the LC-MS analysis. The top 20 pathways with the smallest *p* values are shown by performing the analysis of KEGG pathway enrichment. The y-axis represents the pathway category, and the x-axis represents the degree of enrichment of the individual pathways. The color of the circle indicates the *p* value, and the size of the circle indicates the number of involved metabolites. From red to green indicates the *p*-value level.

**Figure 4:**
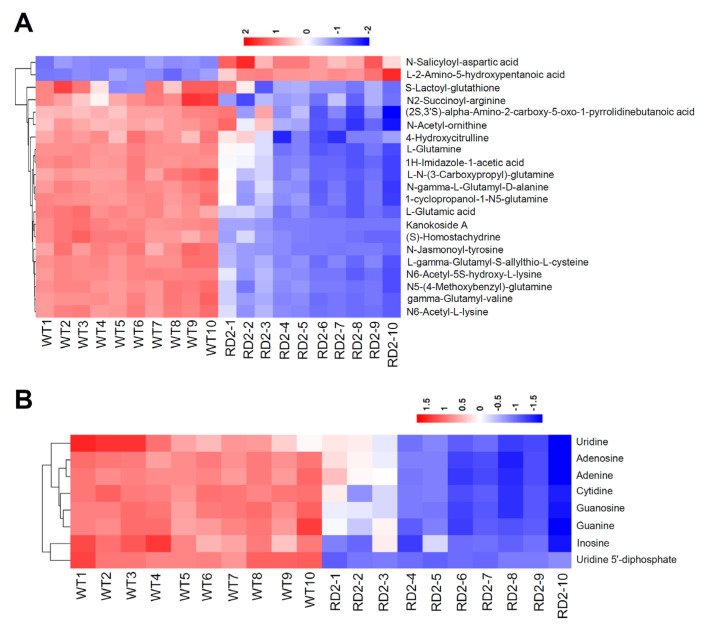
Heatmap of differential clusters of nucleotides and amino acids identified in the flag leaves of WT and transgenic rice line RD2. The cluster heatmap showed significant changes in the levels of amino acids (**A**), purines, and pyrimidines and (**B**) analyzed using LC-MS in the flag leaves of RD2 compared with that of WT. Fold changes are visualized using a color gradient from red (high) to blue (low). Ten biological replicates, taken from the flag leaves of 10 groups of plants of WT and transgenic rice line RD2, are shown.

Based on the KEGG enrichment analysis, several pathways have been enriched among 361 differential metabolites identified in the flag leaves of the RD2 transgenic line using LC-MS. As shown in Figure 3, among the top 20 enriched pathways, a large number of the pathways are involved in the biosynthesis and metabolism of nucleotides and amino acids, such as purine metabolism, pyrimidine metabolism, arginine biosynthesis, nitrogen metabolism, alanine, aspartate, glutamate metabolism, and glutathione metabolism, which were significantly enriched. Given that the metabolisms of purine, pyrimidine, and amino acid were highlighted in the KEGG enrichment analysis, the hierarchical cluster analysis was further performed among these metabolites. As shown in Figure 4A, the relative abundances of 21 differential amino acid metabolites identified using LC-MS were comparatively displayed. Most of the identified amino acids and amino acid derivatives declined in the RD2 plants compared with WT. Among 21 differential metabolites identified using LC-MS, 19 metabolites were significantly reduced in the flag leaves of RD2 compared with WT, including amino acids such as glutamine and glutamic acid, and amino acid derivatives such as L-N-(3-Carboxypropyl)-glutamine, L-gamma-glutamyl-S-allylthio-L-cysteine, N5-(4-Methoxybenzyl)-glutamine, N-gamma-L-glutamyl-D-alanine and N6-acetyl-L-lysine (Figure 4A and Figure S3A,B). For the nucleotides, eight differential metabolites identified using LC-MS were concentrated on purines, including adenosine, guanosine, adenine, guanine, and inosine (Figure 4B), and pyrimidines, including cytidine, uridine, and uridine 5′-diphosphate (Figure 4B). All eight metabolites were remarkably reduced in the RD2 plants compared with WT (Figure 4B and Figure S3C–E).

### 2.4. A Dual Accumulation Pattern of Flavonoids Detected in the RD2 Transgenic Line 

**Figure 5:**
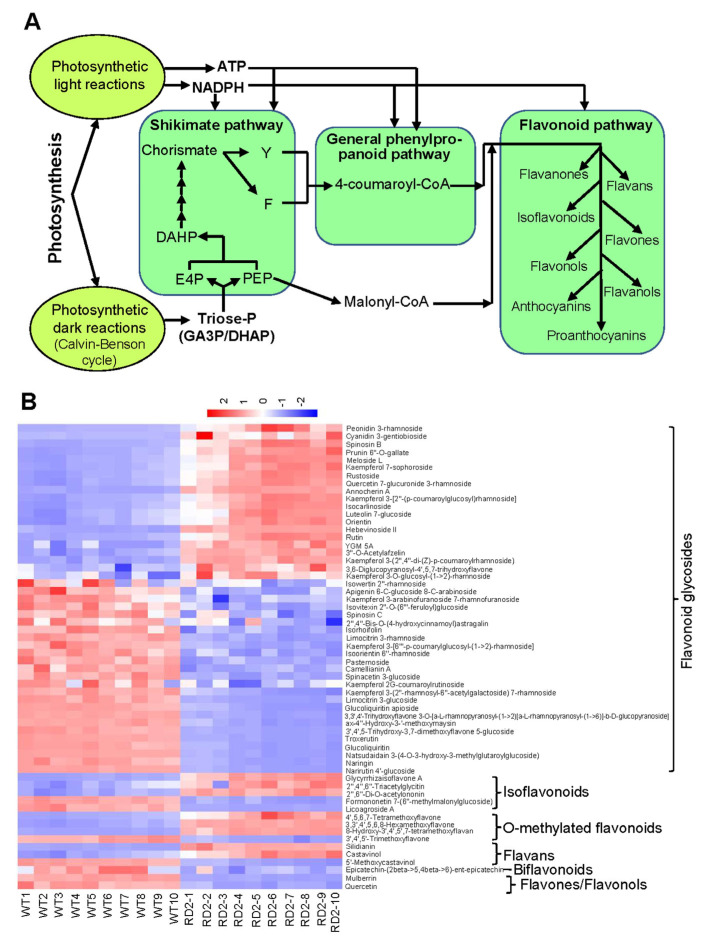
Differential flavonoid metabolites were identified in the flag leaves of RD2. (**A**) The simplified schematic representation of the tight relationships between photosynthesis and the biosynthetic pathways of flavonoids. Photosynthetic light reactions provide NADPH and ATP consumed in flavonoid biosynthesis, and photosynthetic dark reactions (Calvin–Benson cycle) supply the triose-P (GA3P/DHAP) as the primary carbon skeleton substrates in the biosynthetic pathway of flavonoids. DAHP, 3-deoxy-d-arabino-heptulosonate 7-phosphate; DHAP, dihydroxyacetone phosphate; E4P, erythrose 4-phosphate; F, phenylalanine; GA3PD, glyceraldehyde 3-phosphate; PEP, phosphoenolpyruvate; Y, tyrosine. (**B**) The differential flavonoid metabolites identified using LC-MS in the flag leaves of RD2 in comparison with WT are represented as the cluster heatmap. Fold changes are visualized using a color gradient from red (high) to blue (low). Ten biological replicates, taken from the flag leaves of 10 groups of plants of WT and transgenic rice line RD2, are shown.

As one of the most abundant biological pigment classes and the best-investigated classes among the plant secondary metabolites, flavonoids of different structures and functions have been identified in over 8000 in a variety of plant species [25]. Common features have been characterized for all flavonoids, including the presence of a chroman moiety, marked as possessing a substituted phenyl moiety at positions 2, 3, or 4, respectively. In general, all flavonoids are classified into different groups according to the position of the specified fragment with or without a carbonyl group at position 4 of the pyrene ring, a double bond between positions 2 and 3, as well as a positive charge [25,26]. Flavonoids are biosynthesized from phenylalanine and malonyl-CoA by consuming NADPH and ATP, generated using photosynthetic light reactions and using the triose-P (GA3P/DHAP), formed in the Calvin–Benson cycle, as the primary carbon skeleton substrates in the biosynthetic pathway (Figure 5A) [27,28]. With respect to structural features, diphenylpropane (C6–C3–C6) backbone represents a common pattern in which two benzene rings (A ring and B ring) are linked via a three-carbon chain whereby, most flavonoids have been divided into several classes named flavones, flavonols, flavanones, flavanols, anthocyanidins, and isoflavones [28,29]. As one of the major decorations for metabolite modifications, glycosylation occurs in a variety of physiological processes to generate modified natural products, including flavonoids. It has been known that the glycosyltransferases (GTs) enzymes are responsible for catalyzing the glycosylation modification, in which the majority of the sugars naturally can be attached to flavonoids being glucose moieties, and the moieties have also been reported as galactose, xylose, rhamnose, arabinose and glucuronic acid [30,31].

As shown in Figure 5B, the contents of 20 flavonoid glycosides such as rutin, orientin, luteolin-7-glucoside, kaempferol-7-sophoroside, peonidin-3-rhamnoside and quercetin-7-glucuronide-3-rhamnoside were substantially increased in the flag leaves of the RD2 plants, whereas the contents of 25 flavonoid glycosides such as troxerutin, isorhoifolin, naringin, camellianin A, narirutin 4′-glucoside, spinacetin-3-glucoside, and apigenin-6-C-glucoside-8-C-arabinoside were significantly decreased when compared with WT. In addition, the increased levels of isoflavonoids differentially detected in the flag leaves of the RD2 plants included glycyrrhizaisoflavone A, 2″,6″-Di-O-acetylononin and 2″,4″,6″-triacetylglycitin in contrast to the reduced levels of formononetin-7-(6″-methylmalonylglucoside) and licoagroside A in the flag leaves of the RD2 plants (Figure 5B). With respect to O-methylated flavonoids, 4′,5,6,7-tetramethoxyflavone and 3,3′,4′,5,6,8-hexamethoxyflavone and 8-hydroxy-3′,4′,5′,7-tetramethoxyflavan were detected to be increased, whereas 3′,4′,5′-trimethoxyflavone was reduced in the flag leaves of the RD2 plants (Figure 5B). For flavans, two metabolites, including silidianin and castavinol, were detected to be increased, and 5′-methoxycastavinol was detected to be reduced in the flag leaves of the RD2 plants (Figure 5B). Epicatechin-(2 beta->5,4 beta->6)-ent-epicatechin belonging to biflavonoids, mulberrin belonging to flavones, and quercetin belonging to flavonols were measured to be reduced in the flag leaves of the RD2 plants (Figure 5B). These results underpin that supplementing D1 from nuclear origin for enhancing photosynthetic efficiency can have far broader cellular metabolic reprogramming and impact than previously expected. 

### 2.5. Starch as the Primary Product of Photosynthetic Carbon Assimilation Increases in Three Transgenic Lines of Rice 

The Calvin–Benson cycle (CBC) is a photosynthetic carbon reduction cycle in which most of the CO_2_ on Earth can be fixed into organic matter with the energy from sunlight [32,33,34]. For photosynthetic assimilation, glucose-6-phosphate (G6P) is formed via the reaction catalyzed using phosphoglucose isomerase (PGI) from the photosynthetic product fructose-6-phosphate (F6P), and the resulting G6P is further used to generate ADP-glucose in the reactions catalyzed using phosphoglucomutase (PGM) and ADP-glucose pyrophosphorylase (AGPase). For starch biosynthesis, ADP-glucose is linked to the developing chains of starch by starch synthases [35,36,37]. 

**Figure 6:**
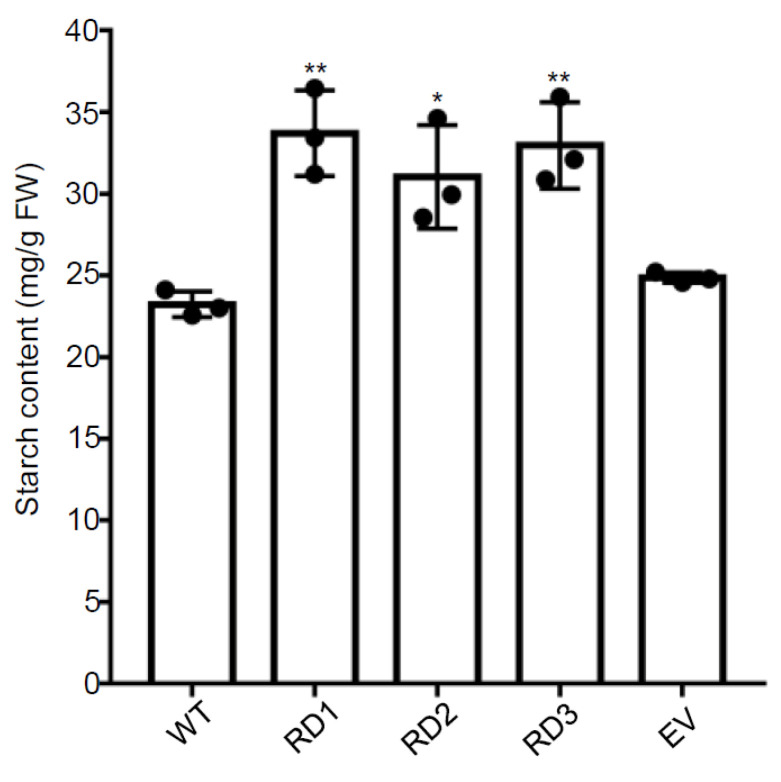
The contents of starch are enhanced in the flag leaves of three transgenic lines of rice. The contents of starch were measured in the flag leaves of WT and three transgenic lines of rice (RD1, RD2, and RD3) (n = 3). The transgenic rice line harboring the empty vector (EV) was taken as a negative control. Individual values (black-coded dots) and means are shown. Bars indicate the SD. * *p* < 0.05, ** *p* < 0.01, two-sided Student’s *t*-test.

In this study, we found that the content of starch significantly increased in the flag leaves of three transgenic lines of rice (RD1, RD2, and RD3) relative to WT and the transgenic rice line harboring the empty vector (EV), which was taken as negative control (Figure 6). These results represent one of the significant changes in three transgenic rice lines, which confers the enhanced photosynthetic assimilation rate, biomass, and grain yield by supplementing D1 with the created nuclear-encoded biosynthesis pathway. The results showed that all the contents of starch in three transgenic lines increased in the range of 33.6–45.1% relative to that in the flag leaves of the WT rice plants (Figure 6). These results indicate that the enhancement in photosynthetic performance by supplementing the PSII core subunit protein D1 results in dramatic accumulation of starch in rice, which can be translated into a significant increase in biomass and grain yield in rice. 

## 3. Discussion

In general, the inorganic forms of nitrogen sources, such as nitrate or ammonium, are acquired by plant roots from the surrounding soil and then assimilated in roots or leaves as amino acids. As the principal nitrogen compounds, amino acids can be utilized within the source organs where they have been assimilated or transported from source to sink organs to support growth and development in higher plants [38,39]. During different growth and developmental stages, the accumulating patterns and compositions of free amino acids in leaves significantly fluctuate, which determines endogenous levels of free amino acids, especially in response to environmental stresses in higher plants [40,41]. 

In this study, our results reveal that leaf-stored nitrogen pools of glutamate and glutamine are substantially reduced in the flag leaves of the transgenic rice line RD2 that harbors the integrated transgene *RbcS^PTP^-OspsbA* cDNA (Figure 4A and Figure S3A,B). In most legumes and crop plants, glutamine and glutamate are the dominant components in the total free amino acid pool, which serve as important nitrogen carriers in plants [42]. Given that the biomass and grain yield of three transgenic rice lines, including RD1, RD2, and RD3, increased significantly with the supplementation of the OsD1 protein using the created nuclear-encoded pathway, the most reasonable explanation for the reduction in the levels of glutamate and glutamine could be that the stimulated growth driven by the enhanced photosynthetic efficiency in the RD2 plants needs consuming more leaf-stored nitrogen sources such as glutamate and glutamine. In rice, glutamine is the major form of reduced nitrogen that is transported through the phloem during nitrogen remobilization from source organs to sink organs [43,44]. In wheat, glutamine was detected as the predominant free nitrogen source at the final stage of grain filling, whereas glutamate is the most abundant amino acid in the phloem of young vegetative plants [45,46]. Extensive studies have been focusing on the tight and coupling relationship between photosynthesis and the availability of nitrogen sources, especially for leaf-synthesized amino acids, transiently stored as nitrogen sources to support rapid growth and reproductive development in plants [47,48,49]. In source organs such as mature leaves, leaf-synthesized amino acids can be loaded into the phloem and transported to growing sinks such as nascent leaves and developing seeds [50,51,52,53,54]. Therefore, our findings suggest that the availability of leaf-stored nitrogen pools such as glutamate and glutamine is likely a key factor influencing the enhanced photosynthetic efficiency, biomass, and grain yield here tested in the RD2 plants under field growth conditions. 

Notably, the reduced levels of glutamate and glutamine also correlate with the remarkable reduction in the levels of purine nucleotides such as adenosine, guanosine, adenine, guanine and inosine, and pyrimidine nucleotides, including cytidine, uridine and uridine 5′-diphosphate in the RD2 plants compared with WT (Figure 4B and Figure S3C–E). In general, the important roles of purine nucleotides such as adenosine, guanosine, and pyrimidine nucleotides such as cytidine and uridine can be explained using the demand for the biosynthesis of RNA and DNA, particularly when the rapid growth and development occurs in developing seeds at the stages of embryo and endosperm development and the establishment of photosynthesis in young seedlings [55,56,57,58,59]. In addition to being blocks for building nucleic acids, nucleotides also act as energy transmitters involved in many other metabolic pathways as well as enzymatic cofactors for biosynthesizing sugars and polysaccharides, glycoproteins, and phospholipids [57,59]. Thus, one could imagine a scenario where the higher demands for leaf-stored nitrogen pools of glutamate, glutamine, and nucleotides for the biosynthesis of RNA and DNA act in concert to support the stimulated growth and increased grain yields as exhibited in the RD2 plants relative to the WT plants. In line with this, the enhanced photosynthetic assimilation capacity in the RD2 plants needs coupling the sufficient nitrogen supply, especially for leaf-stored free nitrogen pools, to fully translate the higher photosynthetic efficiency into the remarkable increase in biomass and grain yields in rice. 

In plants, phenylalanine generated by the shikimate pathway is not only required for the synthesis of proteins but also for secondary metabolites such as flavonoids, and various stimuli like light, pathogens, and wounding play dominant roles in regulating the abundance and diversity of flavonoids [25,28]. A tight relationship exists between the biosynthesis of flavonoids and photosynthesis as the photosynthesis-generated energy and redox strength (NADPH and ATP) and the primary carbon skeleton substrates formed in the Calvin–Benson cycle are used in the flavonoid biosynthesis in plants [27,28]. In this study, our results indicate that the enhanced photosynthetic efficiency, as evidenced in the RD2 plants, induces metabolic reprogramming in the biosynthesis of flavonoids and allows an increased accumulation in certain metabolites of flavonoid glycosides, mainly including anthocyanin (cyanidin and peonidin), quercetin glycosides such as rutin and quercetin 7-glucuronide 3-rhamnoside and other glycosyl flavones (Figure 5B). Despite a range of variations among different plant species, it is estimated that around 20% of the fixed carbon by photosynthesis flows through the shikimate pathway [60]. In accordance with the enhanced capacity of carbon fixation as evidenced in the RD2 plants, the fixed carbon flow through the shikimate pathway could be higher to improve the biosynthesis of flavonoids as detected in the RD2 plants. Actually, a significant increase in the level of starch as the primary product of photosynthetic carbon assimilation was detected in three transgenic lines, including the RD2 plants (Figure 6), supporting the hypothesis mentioned above. According to accumulated data, flavonoids, synthesized by the shikimate pathway, have been suggested to act as a sink for reduced carbon and can also be taken as an energy escape valve by consuming trioses phosphate, ATP, and NADPH generated using photosynthesis [61]. It should be noted that the biosynthesis of flavonoids specifically facilitates the diversion of photoassimilate and energy because it requires the incorporation of malonate, thereby without bearing nitrogen in their chemical structure, which definitely dose not bring an additional pressure on nitrogen resources in plants, especially under abiotic stress or various nitrogen limitation conditions [25,28,61]. 

In white clover, the accumulation of quercetin glycoside was detected with a dramatic increase under drought stress [62,63] and by exposure to UV-B radiation [64]. In tomato plants, the level of quercetin glycoside substantially increased under drought stress [65]. In the different *Amaranthus* species, rutin was identified as the most abundant flavonoid compound when tested under drought and heat stress conditions [66]. The elevated level of rutin was detected in NaCl-stressed cell suspension cultures from *Haplophyllum virgatum* [67]. In rice, the accumulation of rutin was detected under drought stress, conferring the ability to maintain redox homeostasis [68]. As natural water-soluble pigments, anthocyanins, belonging to the large class of flavonoid compounds, accumulate mainly in leaves and stems with various levels in response to a variety of environmental factors such as low or high temperature, drought, high light as well as nutrient starvation [25,28,29,69,70]. Based on previous findings and our results, we propose that the biosynthesis of flavonoids, mainly marked as anthocyanin and quercetin glycosides, may represent an alternative pathway for photochemical energy dissipation, which consequently confers the added benefit of improving the antioxidant capacity in plants. Intriguingly, substantial amounts of flavonoid compounds were also detected to be reduced in the RD2 transgenic plants (Figure 5B). The reasonable explanations for these different accumulation patterns, as mentioned above, could be attributed to the diversity in the sites of flavonoid biosynthesis storage and also the diverse functions of flavonoids at the subcellular, cell, and even tissue and organ levels in plants [71,72,73]. On the other hand, the different capacities of the transport systems for flavonoids across membranes are likely to influence the distribution and accumulation patterns of different flavonoid compounds in plants [73]. 

Taken together, our findings suggest that the availability of leaf-stored free nitrogen sources such as glutamate and glutamine really matters for fully translating the enhanced photosynthesis into biomass and grain yield in crop plants based on our studies on metabolome profiling of transgenic rice plants with increased photosynthetic efficiency by creating a nuclear-encoded synthesis of the PSII subunit OsD1. Therefore, the content of leaf-free nitrogen sources represents a useful marker to incorporate photosynthesis into models of elucidating the relationship between nitrogen and photosynthetic capacity among the species of crop plants.

## 4. Materials and Methods

### 4.1. Plant Materials and Growth Conditions 

The wild type (*Oryza sativa* japonica variety Zhonghua11) and transgenic lines of rice were grown in an isolated paddy field at the Songjiang experimental station of our institute in Shanghai, China. 

### 4.2. Plasmid Constructions and Plant Transformation

To generate the nuclear-encoded *OspsbA* expression construct, driven by the *HsfA2* (AT2G26150) promoter, the amplification of the genomic fragment of the Arabidopsis *HsfA2* promoter region starting 2-kb upstream of the ATG codon was performed using PCR with the primer pair pHsfA2-F and pHsfA2-R, and the resulting fragment was confirmed using sequencing. The resulting promoter fragment was cloned into *SalI* and *BamHI* cloning sites of the expression cassette of pCAMBIA1300. Next, using the primer pair RbcS-PTP-F and RbcS-PTP-R, the amplification of the plastid-transit peptide sequence (PTP) from the *RbcS* gene was performed using PCR [74] and the resulting RbcS-PTP-fragment was digested with *BamHI* and *SmalI* and then cloned into the proHsfA2-pCAMBIA1300 construct, yielding the construct proHsfA2-RbcSPTP-pCAMBIA1300. The mature full-length cDNA of the rice chloroplast gene OspsbA (LOC_Osp1g00110), encoding OsD1, was amplified using PCR with the primer pair psbA-cDNA-F and psbA-cDNA-R, and then ligated to proHsfA2-RbcSPTP-pCAMBIA1300 digested with *SmaI* and *KpnI*, generating the proHsfA2-RbcSPTP-OspsbA-cDNA-pCAMBIA1300 construct for plant transformation. The primer sequences used in this study are listed in Supplementary Table S1. The resulting construct was introduced into *A. tumefaciens* strain EHA105. Transgenic plants of rice were generated as described previously [75]. Homozygous transgenic (T2) plants were screened and confirmed using examining seed germination on the growth medium containing the corresponding antibiotic in combination with PCR analysis. 

### 4.3. Measurements of Net CO_2_ Assimilation Rate

A portable gas exchange system LI-6400XT (LI-COR Biosciences, Lincoln, NE, USA) was used to measure net CO_2_ assimilation rates. For measuring the photosynthetic capacity, measurements were performed on the flag leaves of wild-type and transgenic rice plants at the grain filling stage under 1000 μmol photons m^−2^s^−1^ with 400-µbar CO_2_ supplied using the built-in CO_2_ injection system. The maintenance condition for the cuvette was set to 30 °C for a leaf temperature under a CO_2_ concentration of 400 µmol mol^−1^ and 55% relative humidity.

### 4.4. Analysis of Biomass and Grain Yield under Field Conditions

For conducting a comparative analysis of biomass and grain yield, field experiments were performed in the paddy fields at the Songjiang experimental station of our institute in Shanghai. The plots for cultivating the wild-type and transgenic rice plants were laid out in a randomized complete block design, for which three replicates were performed in paddy fields with plots of 36 plants arranged in 6 rows of 6 plants each. Routine management practices such as irrigation and protection from pests were applied for the plants during growth and development to ensure meaningful field tests. After harvesting, the dry mature plants and seeds were kept at 50 °C for 1 week, and then the above-ground biomass and grain yield per plot were quantified.

### 4.5. Sample Preparation for LC-MS Analysis

For LC-MS analysis, flag leaves (5 g) for each sample were harvested at the grain-filling stage and frozen immediately in liquid nitrogen. For each sample, 80 mg leaf powder was transferred to a 1.5 mL Eppendorf tube. For each sample, 20 μL of internal standard L-2-chlorophenylalanine (0.06 mg mL^−1^) together with 800 μL mixture of methanol and water (7/3, *v/v*) were added. Then, the samples were placed at −20 °C for 2 min and ground at 60 HZ for 2 min. After being extracted by ultrasonic for 30 min in the ice-water bath, the samples were placed at −20 °C for overnight. After centrifuged for 10 min at 4 °C (13,000 rpm), 150 μL supernatants from each tube were collected and filtered through 0.22 μm microfilters, then transferred to LC vials.

### 4.6. LC-MS Analysis

ACQUITY UPLC I-Class system (Waters Corporation, Milford, NH, USA) coupled with VION IMS QTOF Mass spectrometer (Waters Corporation, Milford, NH, USA) was used to analyze the metabolic profiling in both ESI positive and ESI negative ion modes. For both positive and negative modes, an ACQUITY UPLC HSS T3 column (1.8 μm, 2.1 × 100 mm) was employed. Water and Acetonitrile/Methanol 2/3 (*v*/*v*), both containing 0.1% formic acid, were used as mobile phases A and B, respectively. Linear gradient: 0.01 min, 5% B; 2 min, 5% B; 4 min, 30% B; 8 min, 50% B; 10 min, 80% B; 14 min, 100% B; 15 min, 100%, 15.1 min, 5% B. 16 min, 5% B. The flow rate was 0.35 mL min^−1,^ and the column temperature was 45 °C. All the samples were kept at 4 °C during the analysis. The injection volume was 2 μL. The QCs were injected at regular intervals throughout the analytical run to provide a set of data from which repeatability can be assessed. The original LC-MS data were processed using the software Progenesis QIV2.3 (Nonlinear, Dynamics, Newcastle, UK) for analysis.

### 4.7. Data Preprocessing and Statistical Analysis for Metabolome

By using the software Progenesis QI V2.3 (Nonlinear, Dynamics, Newcastle, UK), the original LC-MS data were processed for baseline filtering, peak identification, integral, retention time correction, peak alignment, and normalization. The main parameters are as follows: 5 ppm precursor tolerance, 10 ppm product tolerance, and 5% production threshold. The compounds were identified by using the Plant Metabolome Database (PMDB), Human Metabolome Database (HMDB), and self-built databases for comparison based on the precise mass-to-charge ratio (*m*/*z*), secondary fragments, and isotopic distribution.

Then, the extracted data were further analyzed. Any peaks with a missing value (ion intensity = 0) in more than 50% of groups were removed, and the zero value was replaced by half of the minimum value. By screening the qualitative compounds according to the qualitative results, compounds with resulting scores less than 36 (out of 60) points were also removed as they were regarded to be inaccurate. A data matrix was combined from the positive and negative ion data, and the matrix was imported in R to carry out Principle Component Analysis (PCA) and Orthogonal Partial Least-Squares-Discriminant Analysis (OPLS-DA).

To rank the overall contribution of each variable to group discrimination, Variable Importance of Projection (VIP) values obtained from the OPLS-DA model were used. A two-tailed Student’s *t*-test was further applied to compare the significant differences between groups. Differential metabolites were determined with both multivariate and univariate statistical significance (VIP values greater than 1.0 and *p*-values less than 0.05). These differential metabolites were then selected for subsequent analysis. According to the annotations presented in HMDB and PMDB, all of the differential metabolites are divided into 12 classes. The nucleotides, amino acids, and flavonoids were selected for further cluster heatmap analysis and were performed using the OECloud tools at https://cloud.oebiotech.com. The metabolomic platform was provided by Luming Biological Technology CO., Ltd. (Shanghai, China).

### 4.8. Measurements of Starch Contents

Flag leaves (5 g) for each sample were harvested at the grain-filling stage and frozen immediately in liquid nitrogen. 100 mg of leaf powder for each sample was used for further analysis. Starch content was determined using the Starch Content Assay Kit (Solarbio, Beijing, China) according to the manufacturer’s instructions. The samples were detected at 620 nm by the BioTek Synergy 2 Multi-Detection Microplate Reader (BioTek Instruments, Winooski, VT, USA).

### 4.9. Quantitative Real-Time RT-PCR 

The qRT–PCR analysis was performed as described previously [18]. Briefly, total RNA was isolated from the flag leaves using the TRIzol reagent (Takara, Dalian, China) according to the manufacturer’s protocol. 1 μg total RNA was taken as templates to produce cDNA using the PrimeScriptTM RT reagent Kit with gDNA Eraser (Perfect Real Time) (Takara). A MyiQ5 single-color Real-Time PCR Detection System (Bio-Rad) was used to perform quantitative real-time PCR. The relative transcript levels were quantified using the comparative threshold cycle method (iQ5 admin, Bio-Rad, Hercules, CA, USA). *OsACTIN1* (LOC_Os03g50885) was used as an internal control. The primer sequences are listed in Supplementary Table S1.

### 4.10. SDS-PAGE and Western Blots

Thylakoid membranes were prepared as described previously [76]. SDS-PAGE and Western blots were performed with the prepared thylakoid membrane samples according to the procedures described [18,76]. The D1 antibody (dilution ratio 1:16,000) (Agrisera, Vännäs, Sweden) was used to detect the protein abundance of OsD1. Equal protein loading was confirmed with the antibody against CF1β (dilution ratio 1:10,000) (Agrisera, Vännäs, Sweden). Reactions were developed using an ECL kit (GE Healthcare, Chicago, IL, USA), and ImageQuant LAS 4000 mini (GE Healthcare, Chicago, IL, USA) was used for detecting signals.

## Data Availability

The original contributions presented in the study are included in the article/Supplementary Files; further inquiries can be directed to the corresponding authors.

## Supplementary Materials

The following supporting information can be downloaded at: https://www.mdpi.com/article/10.3390/plants12163009/s1; Figure S1: Alignments of derived amino acid sequences of the D1 protein homologs; Figure S2: Expressing the rice chloroplast gene *psbA* in the nucleus enhances the D1 abundance; Figure S3: Substantial reduction in the contents of representative amino acids and nucleotides identified in the flag leaves of RD2; Table S1: The primer sequences used in this study.
