# Supplementary material for: Supplementing the Nuclear-Encoded PSII Subunit D1 Induces Dramatic Metabolic Reprogramming in Flag Leaves during Grain Filling in Rice
Source: Plants (Basel). 2023 Aug 21;12(16):3009. doi: 10.3390/plants12163009 (PMC10458752; doi:10.3390/plants12163009)
Supplement: Supplementary file 1 [file plants-12-03009-s001.zip › plants-2555760-supplementary.pdf]

## *Supplementary Materials*

# Supplementing the nuclear-encoded PSII subunit D1 induces dramatic metabolic reprogramming in flag Leaves during grain filling in rice

**Ai-Zhen Sun[1#], Juan-Hua Chen[1#], Xue-Qi Jin[1,2], Han Li[1,2] and Fang-Qing Guo[1*]***

**Correspondence:** Fang-Qing Guo: fqguo@cemps.ac.cn

Supplementary Materials include Supplemental Figures S1-3 and Table S1.

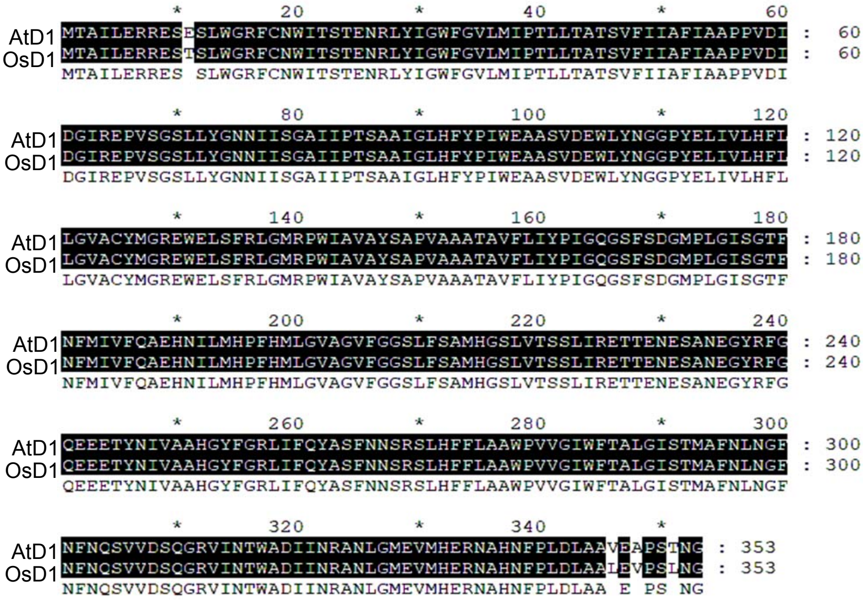


**Supplemental Figure S1**. Alignments of derived amino acid sequences of the D1 protein homologs. Amino acid sequences of AtD1 (NP_051039) and OsD1(AJC09319) were aligned. Identical amino acid residues and conservative changes were depicted in black background.

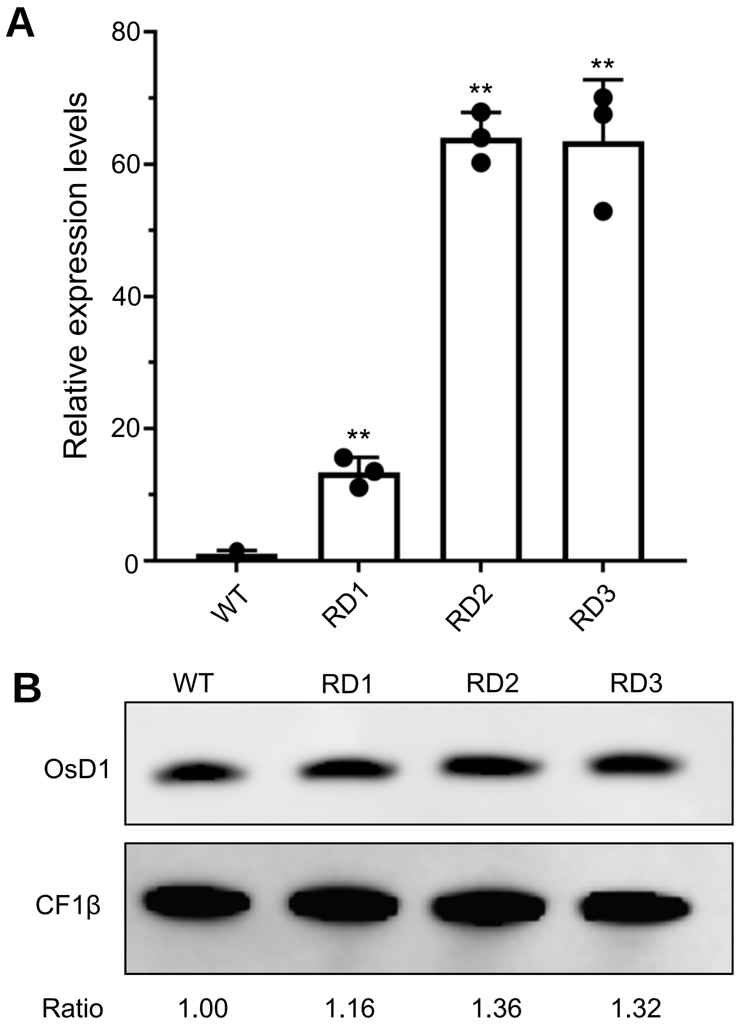


**Supplemental Figure S2.** Expressing the rice chloroplast gene *psbA* in the nucleus enhances the D1 abundance. (A) Relative expression levels of *pHsfA2::RbcS*$^{PTP}$*-psbA* cDNA were analyzed by qRT–PCR in the flag leaves of wild-type (WT) and the transgenic rice lines of RD1, RD2 and RD3. The specific primers RbcS-PTP-qRT-F and OspsbA-qRT-R, derived from the plastid-transit peptide encoding sequence *RbcS-PTP* and coding region of the rice *psbA* cDNA, respectively, were used for analysis. Bars indicate the SD. of three biological replicates. $^{**}P < 0.01$, two-sided Student's *t*-test. Individual values (black-coded dots) and means are shown. (B) Immunodetection of the D1 abundance in thylakoid membranes isolated from the flag leaves of WT and transgenic rice lines of RD1, RD2 and RD3, using the antibody against D1. Equal protein loading was confirmed with the antibody against CF1β. The signal intensities of western blots were quantified using Image J and representative western blots of the protein expression of OsD1 were normalized to CF1β.

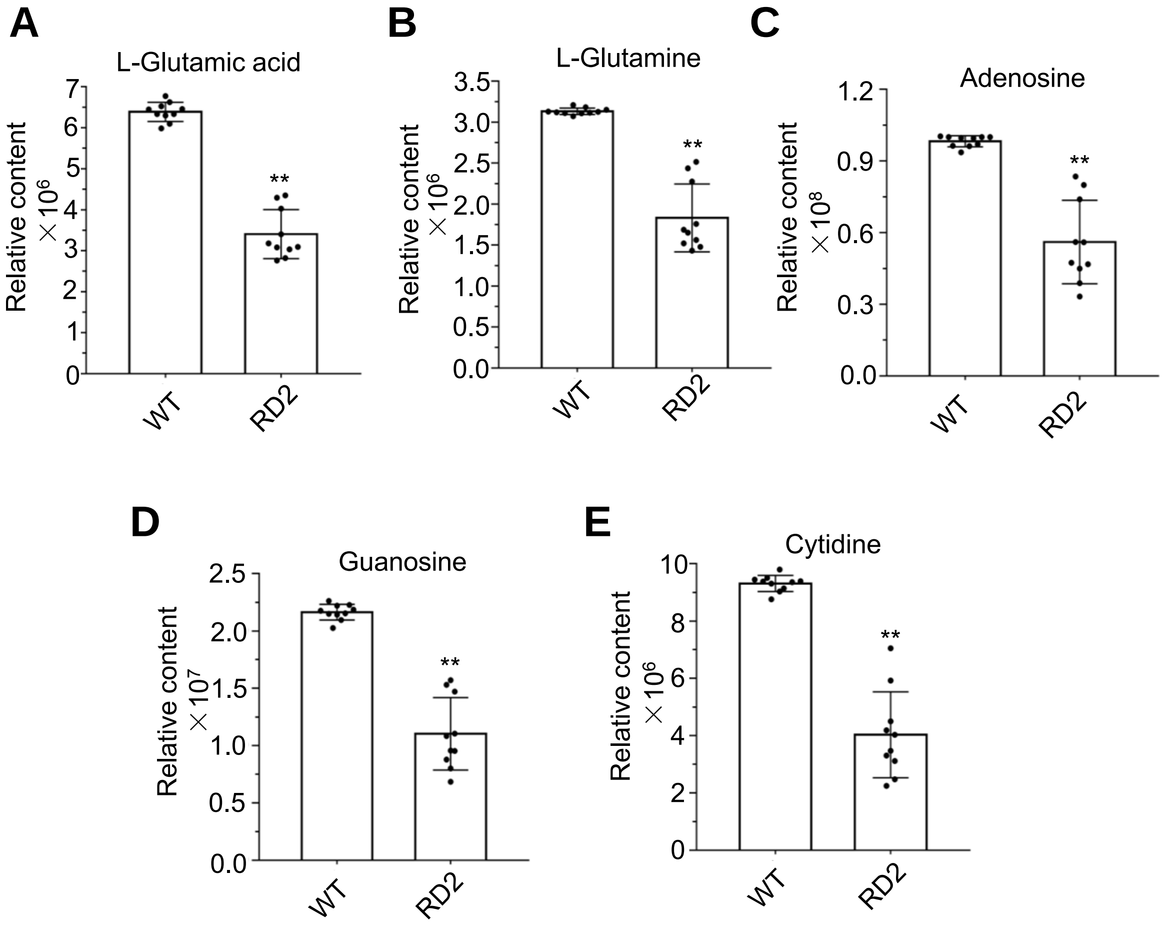


**Supplemental Figure S3.** Substantial reduction in the contents of representative amino acids and nucleotides identified in the flag leaves of RD2. Substantial reduction was detected in the contents of representative nucleotides and amino acids such as L-glutamic acid (A), L-glutamine (B), adenosine (C), guanosine (D) and cytidine (E), identified in the flag leaves of the transgenic line RD2 by the LC-MS analysis in comparison with WT (n = 10). **$P$ < 0.01, two-sided Student's $t$-test.

**Supplemental Table S1. The primer sequences used in this study**

| Primer name | Primer sequence (5’-3’) |
|---|---|
| pHsfA2-F | ACGCGTCGACCTTTGCCAATTCCTCTGTCC |
| pHsfA2-R | CGGGATCCTTTCGTTGTTTATCTCAAAT |
| RbcS-PTP-F | CGGGATCCATGGCTTCCTCTATGCTCTC |
| RbcS-PTP-R | TCCCCCGGGTTCGGAATCGGTAAGGTCAG |
| OsPsbA-F | TCCCCCGGGATGACTGCAATTTTAGAGAG |
| OsPsbA-R | GGGGTACCTTATCCATTAAGAGATGGAACT |
| RbcS-PTP-qRT-F | ATGGCTTCCTCTATGCTCTCTTC |
| OsPsbA-qRT-R | CTCTCTAAAATTGCAGTCAT |
| ACTIN1-qRT-F | TGGTCGTACCACAGGTATTGTGTT |
| ACTIN1-qRT-R | AAGGTCGAGACGAAGGATAGCAT |
